# Postpartum Green Star family planning decision aid for pregnant adolescents in Tanzania: a qualitative feasibility study

**DOI:** 10.1186/s12978-021-01216-6

**Published:** 2021-08-09

**Authors:** Stella E. Mushy, Eri Shishido, Sebalda Leshabari, Shigeko Horiuchi

**Affiliations:** 1 Community Health Nursing Department, School of Nursing, Muhimbili University of Health and Allied Sciences, P.O. Box 65004, Dar es Salaam, Tanzania; 2 Department of Midwifery, Graduate School of Nursing Science, St. Luke’s International University, 10-1 Akashi-Cho, Chuo, Tokyo 104-0044 Japan

**Keywords:** Decision aid, Postpartum family planning, Long-acting reversible contraception, Intrauterine copper device, Implants, Rapid repeat pregnancy, Adolescent mothers, Pregnant adolescents, Tanzania

## Abstract

**Background:**

The use of a decision aid in clinical settings has been beneficial. It informs and educates patients about the available treatment options that can help them reduce decision-making conflicts related to feeling uninformed compared with routine care. There is a scarcity of published data about using a decision aid during family planning counseling with postpartum women focusing on long-acting reversible contraception in Tanzania. Therefore, we developed a *“postpartum Green Star family planning decision aid”* and assessed its feasibility. The study outcomes were practicality, usefulness, and acceptability perceived by pregnant adolescents and nurses/midwives.

**Methods:**

We used an exploratory qualitative in-depth interview involving six nurses/midwives with three or more years of experience in family planning services and 12 pregnant adolescents aged 15–19 years. Purposive sampling was used to select the participants, and selection relied on the saturation principle of data collection. We used a semi-structured interview guide translated into the Kiswahili language. Data were transcribed and analyzed following inductive content analysis.

**Results:**

The amount of information presented was just right, with the time of reading the data ranging from 20 min to 1 h. The study participants perceived the flow of information to be good, with small significant changes suggested. Kiswahili language was used and reported to be appropriate and well elaborated. However, a few words were told to be rephrased to reduce ambiguity. The nurses/midwives said that the decision aid included most of the vital information the participants wanted to know during their family planning counseling. Pregnant adolescents stated that the decision aid improved their knowledge and provided new details on the long-acting reversible contraception methods (intrauterine copper devices and implants) offered immediately after childbirth. The participants stated that the decision aid addressed long-acting reversible contraception methods’ benefits and side effects and dispelled myths and misconceptions. The study participants considered the decision aid helpful in complementing the family planning counseling offered and improving pregnant adolescents’ knowledge.

**Conclusion:**

*The postpartum Green Star family planning decision aid* was practical, useful, and acceptable in enhancing the objectivity of counseling about long-acting reversible contraception methods. It improved the knowledge of pregnant adolescents in Tanzania about the available contraception methods (i.e., the use of intrauterine copper devices and implants), which can be immediately used postpartum. Further research is needed to assess the effects of the decision aid on long-acting reversible contraception postpartum uptake among pregnant adolescents in Tanzania.

## Plain Language Summary

This study assessed the practicality, usefulness, and acceptability of the *decision aid* we developed for pregnant adolescents in Tanzania. The study was conducted at Amana District Hospital in Dar es Salaam, Tanzania. For the study participants, six nurses/midwives with three or more years of experience in family planning services and 12 pregnant adolescents aged 15–19 years were recruited for in-depth interviews using a semi-structured interview guide with five questions. Study participants felt that the decision aid written in the Kiswahili language included important information that women wanted to know during counseling about long-acting reversible contraception options. The amount of information presented was just right, with the time of reading the data ranging from 20 min to 1 h. Study participants recommended that nurses/midwives implement the decision aid to ensure comprehension of the presented information and pictures. The decision aid was perceived to help improve knowledge and address several myths and misconceptions and the benefits and side effects of each long-acting reversible contraception option. Study participants recommended several changes: the flow of information needed reorganization, and some Kiswahili words needed rephrasing to reduce ambiguity. Notably, the study participants accepted the decision aid for use in clinical settings. It contained evidence-based information useful for complimenting the family planning counseling offered, particularly for long-acting reversible contraception methods. The decision aid helped improve the objectivity of counseling regarding long-acting reversible contraception methods. It imparted valuable knowledge to pregnant adolescents in Tanzania about the contraception methods (i.e., the use of intrauterine copper devices and implants) that are immediately available after childbirth. Additional studies are needed to examine the effects of the decision aid on pregnant adolescents in Tanzania to uptake long-acting reversible contraception methods after childbirth.

## Background

Adolescent pregnancies remain a global public health concern, with the highest rate occurring in developing countries. Across the world, about 16 million girls aged 15–19 years are estimated to give birth each year [1], and most of the pregnancies are reported to be unintended [1, 2]. Unintended pregnancies are pregnancies that are unplanned or unwanted [3].

According to the latest Tanzania Demographic Health Survey available [4], the adolescent population (10–19 years old) comprises 23% of the total population (44.9 million). Most adolescents are sexually active before the age of 15 years, and the number of childbearing adolescents aged 15–19 years has gradually increased from 23% in 2010 to 27% in 2016. Likewise, the adolescent fertility rate has increased from 116 to 132 per 1000 girls between 2010 and 2016, and at the age of 19 years, almost 56.7% of adolescents have begun childbearing.

Adolescent pregnancies remain a significant national problem and critical health and social priority in Tanzania [5, 6]. One-quarter (24%) of the pregnancies among young women are unintended [7]. The country statistics show that 21% of young women's pregnancies occur among adolescents aged 15–19 years, of which 18.1% are first births and 2.9% are second or later births [4].

Most women who start childbearing in their teenage years have more children and shorter birth spacing. Furthermore, most of their births are unintended compared with women who began childbearing when they were 20 years or older [8]. Subsequent pregnancies during the teen years compound the economic, physical, and psychological consequences of adolescent mothers [8, 9]. Failure to complete high school and impaired economic sufficiency are significantly associated with repeated pregnancy during adolescent age [10].

Inconsistent use of contraception, lack of knowledge about immediate use of family planning after birth, lack of communication between teens and their healthcare providers, fear of side effects, and myths and misconceptions about family planning increase the risk of unintended adolescent pregnancy and rapid repeat pregnancies (RRP) [11–13]. Weight gain, reduced sexual desire, irregular bleeding, infertility, and abnormal vaginal discharge are described as the main barriers hindering the female youth from going to family planning services [11].

To prevent early pregnancies, countries across the world, including Tanzania, have implemented sexual and reproductive health education, enhanced negotiation skills building, and improved awareness and accessibility to modern contraceptives among adolescents [14]. However, most interventions focus on preventing first pregnancies with fewer efforts to prevent subsequent unintended pregnancies during the teen years. As a result, most adolescents continue to experience rapid repeat pregnancy despite the availability and accessibility of family planning methods. The majority (78%) of non-first pregnancies among adolescent mothers in Tanzania experience rapid repeat pregnancy within three months after birth, with over half of the births (51%) occurring in the second year from the last birth [13]. This is an alert that calls for researchers, healthcare providers, and policymakers to put more effort into supporting pregnant adolescents in the planning and timing of subsequent pregnancies.

Delaying the second birth will provide a better chance for an adolescent mother to mature psychologically and biologically. Ultimately increases her opportunities to complete high school, make plans for the future, and develop other training skills [15]. Delaying the second birth will also reduce the risks of premature births, stillbirths, and newborn morbidity and mortality [16].

The effective use of family planning is one of the primary and essential strategies for reducing high-risk pregnancies, which often occur too early and frequently [17]. The use of long-acting reversible contraception immediately after childbirth (i.e., intrauterine copper devices and implants) is considered a high-impact intervention that reduces the risks associated with adolescent pregnancy [18]. These methods do not rely on daily, weekly, or monthly use and have higher continuation and satisfaction rates than short-acting reversible contraception [18]. Research findings from a previous study found that adolescent mothers who initiated long-acting reversible contraception after delivery had a lower risk of rapid repeat pregnancy than adolescent mothers who started short-acting reversible contraception or no family planning methods [19].

Despite significant gains in the training of healthcare providers, distribution of family planning commodities, and provision of quality of care, the postpartum contraceptive uptake among adolescent mothers is still critically underutilized. Only 12.2% of teenage mothers reportedly used postpartum contraceptives within three months after delivery, the largest proportion using injectables followed by pills [4]. Both intrauterine copper devices and implants are reportedly highly effective in preventing pregnancy, and they all last for several years and are easy to use [1, 18]. For these reasons, pregnant adolescents need comprehensive information about all the contraception options available in their country to help them decide on the contraceptive they can use. In the case of pregnant adolescents in Tanzania, this involves clarifying their values and beliefs and what is important to them, particularly regarding intrauterine copper devices and implants being the only locally available long-acting reversible contraception methods.

Decision aids in clinical settings have been beneficial as they inform and educate patients about the available treatment options, which help reduce decisional conflicts [20]. Recently, decision aids are widely used to provide health information to patients and the public [21]. As intrauterine copper devices and implants have almost equal effectiveness and are long-lasting devices, the choices of pregnant adolescents will depend on their preferences and lifestyle. Pregnant adolescents must clarify their values by appraising the benefits and harms of each option and weighing attributes that are personally important to them when making a choice.

Decision aids on family planning counseling have a positive outcome [22, 23]. But to our knowledge, there is still no study on decision aids that focus on long-acting reversible contraception methods to improve family planning uptake by adolescent mothers immediately after childbirth. To avoid the long-term physiological, psychological, and economic consequences adolescent mothers face from unintended rapid repeat pregnancy due to non-contraception use, we developed a decision aid named *postpartum Green Star family planning*. We borrowed the name “Green Star” from the Kiswahili word “Nyota ya Kijani,” a well-known word to most people in Tanzania. The Tanzanian government launched the National Family Planning project in 1992 and used the Kiswahili word “Nyota ya Kijani” as the project name. The project focused on sensitizing women of reproductive age to use artificial family planning methods to space their pregnancy and limit the number of their children. The postpartum Green Star family planning decision aid that we newly developed includes updated evidence-based information on intrauterine copper devices and implants to guide pregnant adolescents decide regarding the best option for using long-acting reversible contraception methods based on their preference. Its relevance lies in the guidance provided to pregnant adolescents in using artificial family planning methods to space and reduce pregnancy, similarly to the principle of “Nyota ya Kijani.”

In the present study, we assessed the practicality, usefulness, and acceptability of the *postpartum Green Star family planning decision aid* as perceived by pregnant adolescents and healthcare providers in Tanzania when choosing long-acting reversible contraception options offered immediately after childbirth. Specifically, we focused on two long-acting reversible contraception methods available and can be provided within 48 h postpartum in Tanzania.

## Materials and methods

### Study design

The study used an exploratory qualitative research design. In August 2020, we conducted 18 in-depth interviews that involved healthcare providers and pregnant adolescents from Amana District Hospital in Dar es Salaam. Although the decision aid was designed for pregnant adolescents, we included healthcare providers because we wanted to hear their opinions about the decision aid. We believe that the family planning counseling experiences of the healthcare providers would provide significant and well-informed comments to improve the contents of the decision aid and the intervention process.

### Study population, recruitment, and data collection process

The study population consisted of six healthcare providers and 12 pregnant adolescents. The healthcare providers were nurses/midwives with three or more years of experience in family planning services. The pregnant adolescents were 15–19 years of age who could read and communicate in the Kiswahili language and consented to participate in the study. The study's principal investigator and the person in charge of the maternity clinic were responsible for recruiting the study participants who were purposively selected to best answer the research questions. The interviews lasted for 40–50 min and were led by two moderators: one who asked the questions and another who assisted and recorded the interview and took notes. A semi-structured interview guide with five questions in the Kiswahili language was used to collect information from pregnant adolescents and healthcare providers. In the interview, participants were asked the following questions: “What do you think about the information presented in the decision aid?”, “How do you feel about the use of a decision aid for deciding postpartum family planning?”, “What do you think about the usefulness of the decision aid?”, “Will you recommend its use in the clinic?” and several follow-up questions.

We obtained ethical approval from St. Luke's International University, Muhimbili University of Health and Allied Sciences, and the National Institute of Medical Research. Permission to conduct the study was obtained from the regional medical officer and the medical officer-in-charge of Amana District Hospital. The interviews were conducted in a private room within the hospital to ensure convenient access and privacy. The principal investigator informed the participants about the study's purpose, scope, and importance at the beginning of each discussion. The study participants were informed of their rights to participate and withdraw during the interview whenever they felt like it. Written informed consent was obtained from each participant for participating and recording the interview. The principal investigator requested the participants to feel free and be open when responding to questions and assured them that there was no right or wrong answer. The principal investigator and a research assistant conducted the interviews. Experience in conducting interviews, experience in using recorders, having knowledge, and experience in family planning were the criteria used for selecting the research assistant. A one-day training was conducted for the research assistant to comprehensively understand the purpose of the study, what questions to ask, how to ask, and probe and when to obtain written informed consent from the participants not to influence data collection.

### Data processing and analysis

The P.I. uploaded the audio files into a secured computer with a passcode immediately after all interviews each day. The interviews were transcribed verbatim in the Kiswahili language, and the data was analyzed while in the Kiswahili language. We conducted thematic analysis following the steps outlined by Braun and Clarke [24]. An iterative inductive-deductive, the team-based coding approach was employed to code and analyze the data [25]. The P.I. and R.A. who conducted the in-depth interviews undertook the coding process and analysis. Individual codes were then organized into subcategories and categories. study participants’ quotes illustrated the key findings.

Using a team-based approach, we developed the codebook [25] after re-reading all the transcripts (familiarization with data). The P.I. and R.A. had several meetings where codebooks and memos were presented, codes updated, and any existing disagreement was resolved.

Next, the P.I. and R.A. generated themes that involved open-ended coding of several transcripts with no predetermined codes or categories. Coding was done directly onto the hard copies of the transcripts during multiple readings of the interviews. Independent from each other, the P.I. and R.A. coded interviews question by question and then shared and compared their coding findings to reconcile differences, if any. The P.I. and R.A. applied the codes from the codebook to all 18 transcripts. Codes were refined, reduced, and expanded during this period. Finally, the P.I. and R.A. generated categories and subcategories based on the findings of the initial coding. Codes were grouped under categories and subcategories.

#### Development of the postpartum Green Star family planning decision aid

The author identified the gap, target population, and objectives to address the research problem. We mainly focused on previously published studies to determine the objectives to be addressed [21, 23, 26–28]. There are limited publications that described the use of decision aids in reducing decision-making conflicts on the utilization of long-acting reversible contraception methods.

**Fig. 1:**
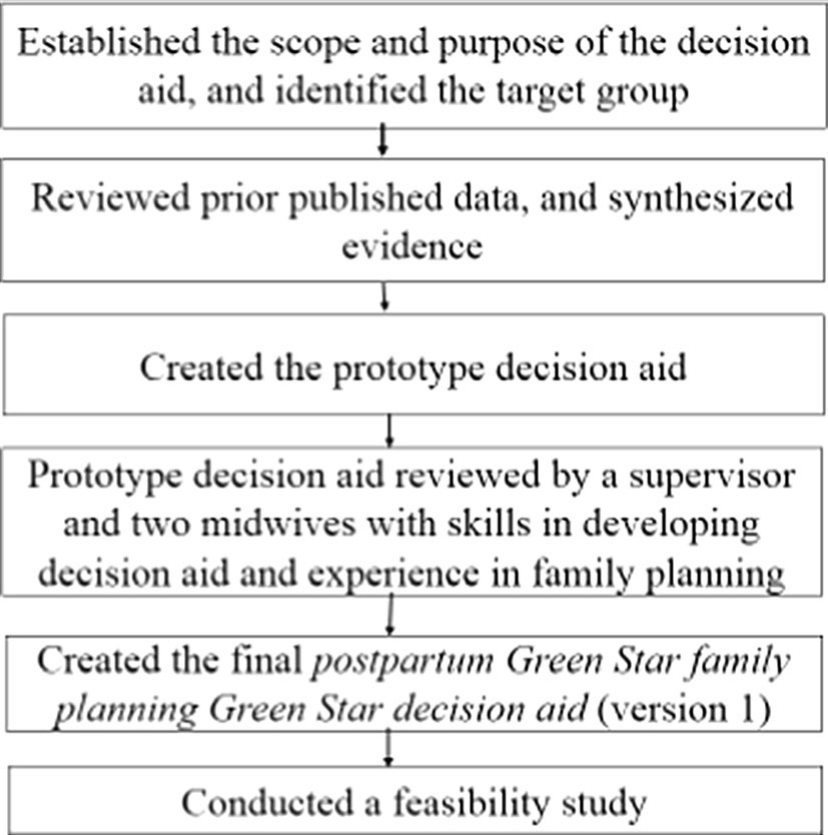
Flow diagram showing the process of developing the final *postpartum Green Star family planning decision aid* (version 1)

We then identified the individual needs of the participating pregnant adolescents by reviewing a previous study that looked at barriers to the utilization of family planning among female youths in Dar es Salaam, Tanzania [11]. The individual needs of the participants included inadequate knowledge, especially of long-acting reversible contraception methods. We found female teens to have several misbeliefs about the methods and how to participate in decision-making. Most female teens could not decide on their own without involving their sexual partners (Fig. 1).

**Fig. 2:**
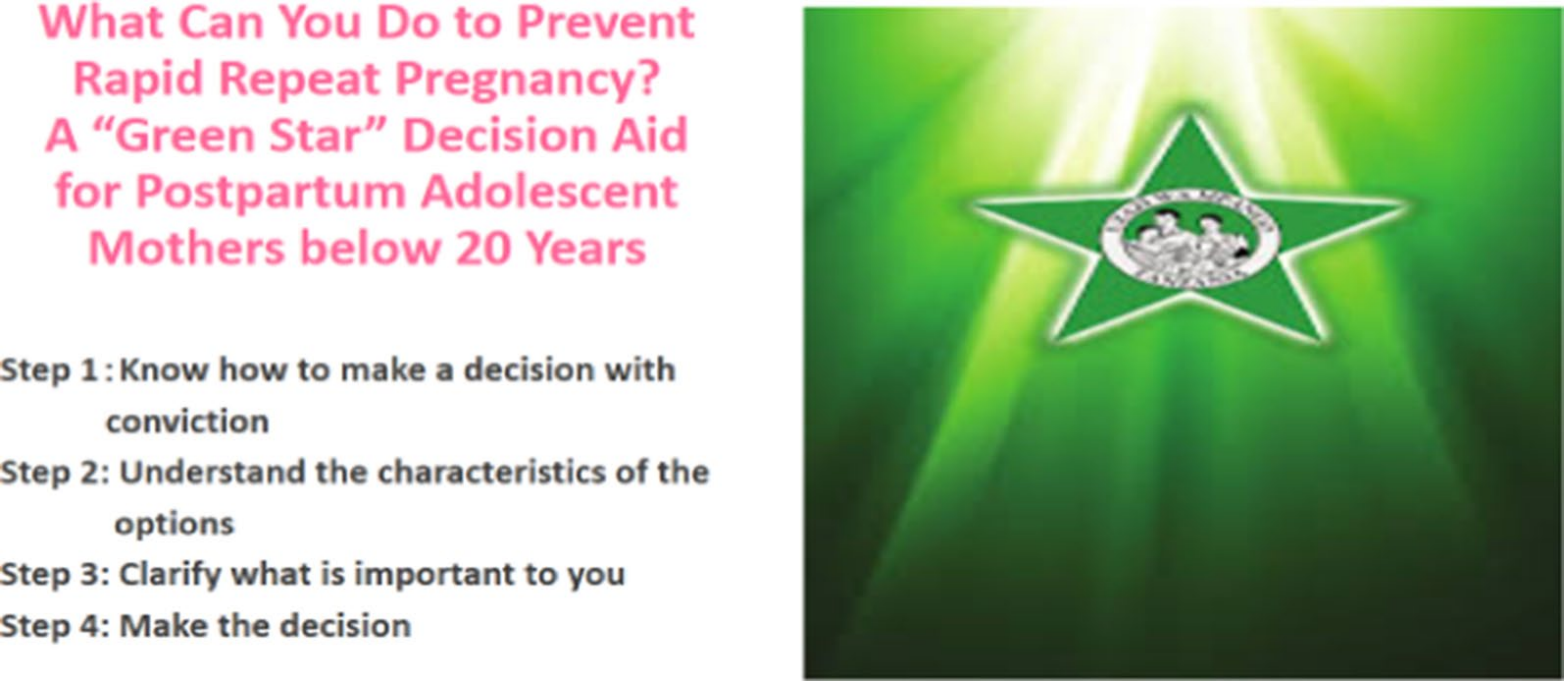
Four steps involved in developing the decision aid based on the Ottawa Patient Decision Aids development

**Fig. 3:**
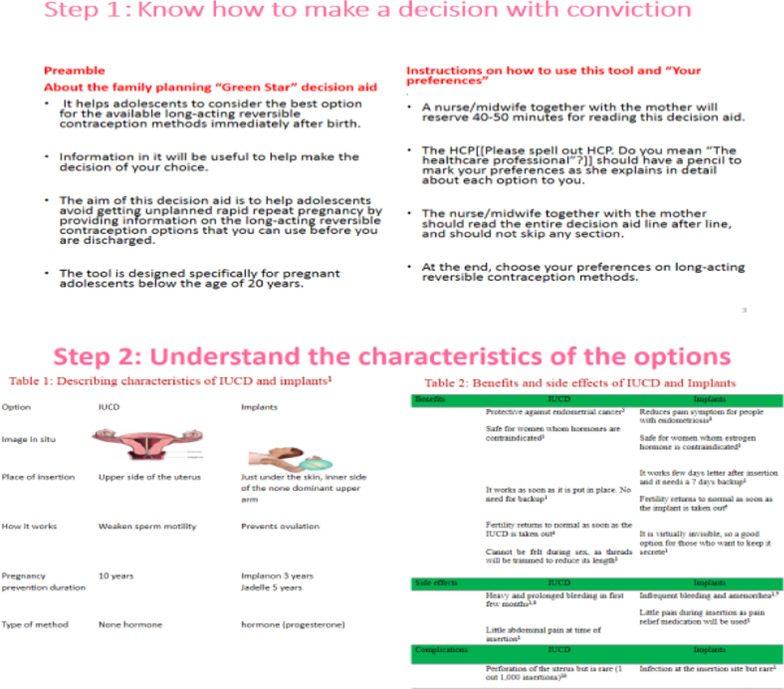
Contents in steps 1 and 2 of the *decision aid*

**Fig. 4:**
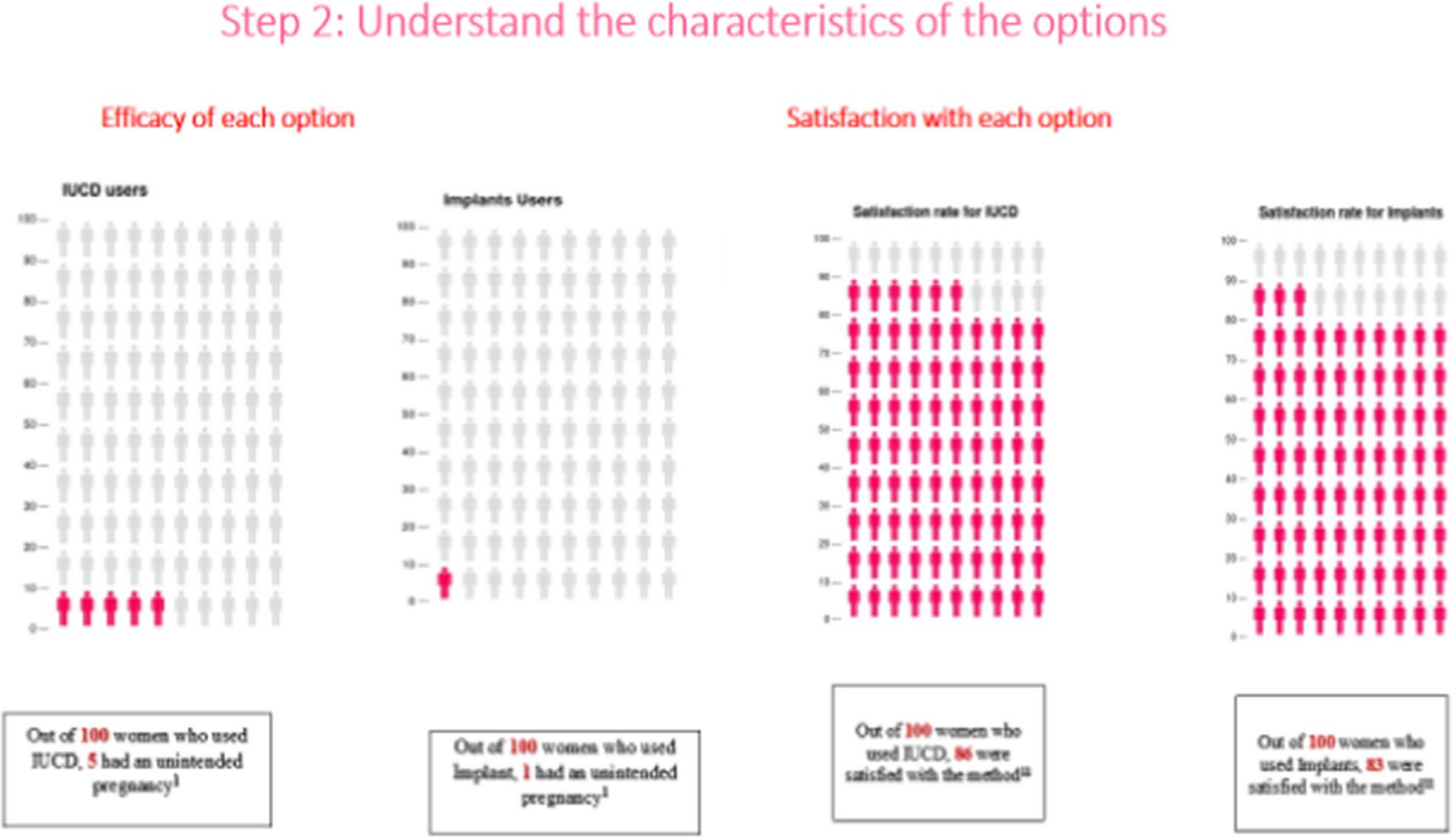
Contents in step 2 of the decision aid: understand the characteristics of the options

**Fig. 5:**
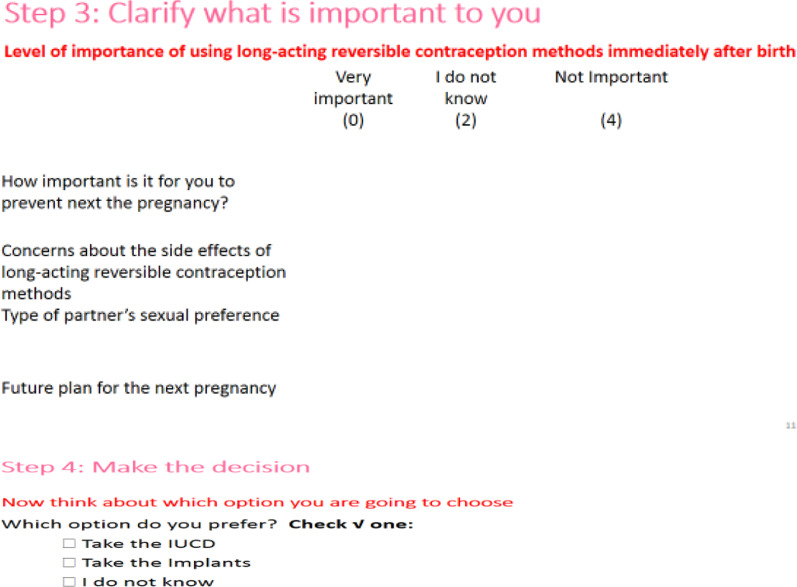
Contents in steps 3 and 4 of the decision aid

The content, design, and arrangement of the “developed prototype decision aid were grounded in the Ottawa Patient Decision Aid Development eTraining [29], International Patient Decision Aid Standards Collaboration checklist [30], Theory of Planned Behavior [31], Health Belief Model [32], Social Cognitive Theory [33], current clinical guides for family planning counseling for providers [1, 34], and findings from previous studies on the benefits and side effects of the options, satisfaction and continuation rates, and fertility return [35–41]. The prototype decision aid has four components based on the Ottawa Patient Decision Aid development guide: (1) know how to make a decision with conviction; (2) understand the characteristics of the decision; (3) clarify what is important to you, and (4) make the decision (Figs. 2, 3, 4, 5). The author shared a prototype decision aid with three experts that involved a research supervisor and two midwives, all with several years of experience in maternal and child health and developing decision aids. The aim of sharing the prototype decision aid with these experts was to receive comments on the comprehensibility and usefulness of the prototype decision aid, which we incorporated to modify and improve the decision aid. The final decision aid version 1 was given to six healthcare providers and 12 pregnant adolescents to assess its practicality, usefulness, and acceptability (Fig. 1).

## Results

### Sociodemographic characteristics of the study participants

We interviewed a total of 18 participants: six healthcare providers (2 male and 4 female midwives) and 12 pregnant adolescents. One healthcare provider had a bachelor’s level of education, and the rest had a master’s level. All the six healthcare providers had family planning service experience of at least three years (i.e., 3–12 years of experience). The majority (83%) of the pregnant adolescents had a primary level of education, more than 58.3% had no employment, and half of them were still single. More than half (66.7%) of the pregnant adolescents reported the index pregnancy to be their first pregnancy.

The present study looked at the practicality, usefulness, and acceptability of the *postpartum Green Star family planning decision aid* in choosing postpartum family planning among pregnant adolescents in Tanzania from the perspectives of both pregnant adolescents and healthcare providers. From the data analysis, three major categories were derived from the questionnaire: (1) practicality, (2) usefulness, and (3) acceptability.

### Practicality

The study participants were asked to discuss their views about the information presented in the decision aid. They looked at the amount *of data,* whether too long, too short, or just right*, the quality of presented information* regarding flow, clarity, and *comprehensibility* of the information and language used. The study participants expressed that the amount of information presented was adequate. The minimum time reported by the pregnant adolescents to finish reading the decision aid was 25 min, whereas the maximum time was 60 min:

*“It took me 20–30 min to read the whole decision aid” [Pregnant adolescent]*

*“I used an hour to read all the information” [Pregnant adolescent]*

The study participants were asked to give their views about the flow of contents, clarity, comprehensibility, and changes they think should be incorporated to improve the decision aid. The study participants expressed that the flow of contents was good, systematically organized, and simple:

*“Generally, the decision aid is good, and the information in it covers the areas that we aspire to be covered in a family planning counseling to be understood by every woman. The flow is good, and I like the decision aid most because it involves pictures with interesting colors that will improve the clarity of the presented information” [Healthcare provider]*

*“To me, I see the information presented as normal as I cannot say it is too long or too short, the explanation is clear and easy to understand” [Pregnant adolescent]*

However, a healthcare provider suggested a small significant change to improve the logical flow of contents. They further said contents included most of the critical information needed when providing counseling and key information/components essential to aid decision-making:

*“Looking at the experience of counseling, there are lots of important aspects presented and often are the things clients ask especially [about] the effectiveness and fertility return. It [decision aid] has also described each option, where it is inserted, and how it works to prevent pregnancy” [Healthcare provider]*

The pregnant adolescents expressed that the language used (Kiswahili) was clear, and everyone who gives time to read the decision aid will understand it because the information was elaborated. Nevertheless, the healthcare providers gave some suggestions to reduce ambiguity, such as the use of the word kitanzi instead of lupu (IUCD) or the use of both; the use of kichocheo instead of the “hormone”; the use of “slows down sperm motility” instead of “killing sperms”:

*“I would say that the information presented in the tool [decision aid] was easy to understand because the Kiswahili words used are simple and well elaborated” [Pregnant adolescent]*

*“There are some words that I thought, if used, would enhance clarity. For example, family planning clients are [more] familiar with the word “kitanzi” than “lupu”. Use Kichocheo and not hormone as that is an English word. Similarly, killing sperms will be strange to women; instead, rephrase it to “slow down the sperm motility” [Healthcare provider]*

The study participants advised that somebody should read the information in the decision aid to the study participants to help them understand the information presented in the tool, especially the colored and uncolored pictures.

*“I recommend the healthcare providers to brief the participants about the information in every step and the meaning before they take the document home for further self-reading due to differences in the level of understanding” [Healthcare provider]*

*“It will be difficult for me to read and understand all the information. When I finish reading the first page and go to the next page, I do not remember what I read on the previous page. I need someone to explain to me as when I hear someone talking; it sticks in my head more than if I read myself [Pregnant adolescent]*

### Usefulness

The study participants were asked if the decision aid would improve knowledge, address the existing myths and misconceptions about intrauterine copper devices and implants in the community, and if each option's benefits and side effects in the decision aid are well clarified. The pregnant adolescents reported that the decision aid improved their knowledge after reading it. Pregnant adolescents said they were not informed if intrauterine copper devices and implants can be used as the immediate postpartum family planning methods. They further stated that they did not know how the family planning methods are put in place, but now they could imagine from the pictures shown in the decision aid:

*“I did not have prior information about intrauterine copper devices and implants apart from pills and injectables, but after reading [the decision aid], I realized [that] there are other family planning methods that even last longer than a year.” [Pregnant adolescent]*

The healthcare providers affirmed that the decision aid would be helpful for pregnant adolescents. They further said that the decision aid explains the types of women, including pregnant adolescents, who can use any of the family planning options, which is missing in most of the family planning counseling sessions and guides:

*“The tool [decision aid] is a narrative that can be read within a short period and be understood than reading the entire family planning book. The tool pointed out people who can use long-acting reversible contraception methods that included women of any age as long as they are sexually active. Most teenagers think that contraceptives are for adults above their twenties. So, this information opens up their mind and helps them realize [that] family planning is for any woman regardless of age as long as she is sexually active. This part has been overlooked in our family planning counseling even in family planning clinical guides” [Healthcare provider]*

The healthcare providers reported having seen some myths and misconceptions addressed in the decision aid. However, they pointed out that these are very few compared with the many existing misbeliefs in the community, especially about the long-acting reversible contraception methods. The healthcare providers pointed out misbeliefs, such as women conceived while an intrauterine copper device was in their uterus. The baby would be holding the intrauterine copper device when it is born. They further said the intrauterine copper devices and implants occasionally escape from where they are and go to the heart or brain. The healthcare providers further reported no specific section in the decision aid that talks about myths and misconceptions. Reading the decision aid alone would not be sufficient to dispel any existing misbeliefs about the family planning methods for pregnant adolescents. When the pregnant adolescents were probed about this part, they all kept quiet despite clarifying the question for them several times:

*“The tool has addressed some of the existing myths and misconceptions indirectly, but they are very few. There are a lot of misbeliefs existing in our communities. If you address more of them, this tool [decision aid] will be useful in addressing the existing misbeliefs” [Healthcare provider]*

The healthcare provider noted that the last part of the decision aid required pregnant adolescents to note down some questions that need more clarification in the next antenatal clinic. This would help explain the concerns raised and include myths and misconceptions a pregnant adolescent had about the family planning methods not described in the decision aid. The Healthcare provider further expressed that it was not easy to address all of the myths and misconceptions existing in the community about intrauterine copper devices and implants as it would make the decision aid too long:

*“The part that might help to cover the question about myths and misconceptions is in the comments section. If the study participants have any ideas or misconceptions about the things they read and need clarifications, I think this part might help. If they have any questions or something they understood differently, writing here for more clarification in the next antenatal clinic visits will help to address the concerns the woman has about family planning” [Healthcare provider]*

The study participants reported that the decision aid clarified each family planning option's benefits and side effects. The safety of artificial family planning was a concern and a barrier to using family planning methods. As the decision aid addressed such matters, it would provide realistic expectations of side effects and later would improve voluntary decision-making to utilize family planning immediately after delivery:

*“Yeah! The area where society is stuck is understanding how contraceptives work, how they prevent pregnancy. If real, they do not cause all the complications (myths and misconceptions) they hear in the communities. The tool described the benefits and side effects that each option can cause. I think this tool [decision aid] will be useful in improving the utilization of family planning” [Healthcare provider]*

*“The tool [decision aid] has been explained well by comparing the benefits and side effects of each option, for example, I have read that the loop does not add on body weight and neither can my partner feel it during intercourse because they reduce the length of the string and this was my biggest fear as I heard it from my sister” [Pregnant adolescent]*

### Acceptability

We asked the study participants if they would recommend the decision aid to encourage women, including adolescents, to use long-acting reversible contraception methods. Two reasons for accepting the decision aid came from the analysis: (a) the decision aid improves knowledge, and (b) the decision aid is a standardized guide with evidence-based information.

The pregnant adolescents expressed that the decision aid should be used as it helped them get information that they were previously unfamiliar with the existence of family planning methods offered immediately after giving birth:

*“Let it [tool] be there in the clinic so that we can use it as it helped me to understand the available postpartum family planning methods offered immediately after birth. I am sure even my fellow women will find the same benefits” [Pregnant adolescent]*

The healthcare providers supported the use of the decision aid to complement their family planning counseling education. They commended the decision aid for being simplified and including all essential evidence-based information, particularly for mothers who need to learn the importance of child spacing, especially in postpartum family planning. The decision aid was seen to be an excellent standardized guide for family planning, specifically for long-acting reversible contraception methods offered immediately after childbirth:

*“Yes! The tool is so simplified, and it included only the important information that a mother needs to be aware of to make an informed choice of the long-acting reversible contraception options. Generally, the tool is good to be used for both mother and caregiver” [Healthcare providers]*

Healthcare providers further expressed that they would recommend using the decision aid because the healthcare facilities do not have a standardized guide that can be used for family planning counseling. They find the family planning counseling offered in healthcare facilities to be subjective. There is no well-structured tool apart from the World Health Organization family planning handbook for healthcare providers [41]. Thus, you find different kinds of information are given to mothers simply because of the healthcare providers' differences in knowledge, time, and attitude. The use of the postpartum Green Star family planning decision aid is formal and is anticipated to enhance the objectivity of information offered to everyone:

*“I will support the use of the tool [decision aid] because the current family planning health education offered is too subjective as there is no standardized guide for family planning counseling. Since this is formal, it will ensure the objectivity of counseling to everyone as mothers will receive the same information from different healthcare providers than when there is no a standardized guide” [Healthcare provider]*

## Discussion

We highlight the following main findings regarding the feasibility of using the *postpartum Green Star family planning decision aid* for pregnant adolescents in Tanzania. First, in terms of the decision aid's practicality, the amount of information presented was just right, as the time needed to complete the reading of the decision aid ranged from 20 to 60 min. The flow of information was good, with small but significant changes suggested by the healthcare providers to improve the logical flow. The Kiswahili language and medical terms used were clear and well elaborated, although study participants suggested minor changes in some words. The pregnant adolescents and healthcare providers recommended that the decision aid should be designed, delivered, and explained by healthcare providers to pregnant adolescents and not just given to pregnant adolescents for self-reading.

*Second*, as for the usefulness of the decision aid, the pregnant adolescents reported that the decision aid improved their knowledge about family planning methods used immediately after childbirth. They have not been aware of intrauterine copper devices and implants until they read the decision aid. The healthcare providers indicated that the decision aid dispelled some existing myths and misconceptions about intrauterine copper devices and implants. The pregnant adolescents and healthcare providers stated that the decision aid addressed each option’s benefits and side effects.

*Third*, about acceptability, the healthcare providers accepted the decision aid as a helpful tool for complementing the family planning counseling offered. They further stated the decision aid would improve the objectivity of the family planning counseling offered about the long-acting reversible contraception methods. The pregnant adolescents expressed that the decision aid improved their knowledge about long-acting reversible contraception, which they lacked until they read the decision aid.

The *developed postpartum Green Star family planning decision aid* in this study is a unique tool designed to potentially address the significant gaps in family planning delivery programs in developing countries. We created the decision aid primarily to be used and evaluated by pregnant adolescents. Still, we also included healthcare providers as we wanted to explore the practicality, usefulness, and acceptability of the decision aid from their perception concerning their experiences from their current counseling practices. Although the decision aid was designed primarily for pregnant adolescents, the results showed that the decision aid was acceptable to pregnant adolescents and healthcare providers. The healthcare providers considered the decision aid to help complement their daily family planning counseling. It also provides objectivity for the counseling, particularly on the long-acting reversible contraception methods. However, the healthcare providers emphasized that they should use the decision aid to guide pregnant adolescents in making an informed decision instead of leaving the decision aid with the pregnant adolescents to read and arrive at a conclusion by themselves.

On the other hand, the pregnant adolescents stated that healthcare providers should guide them through the decision aid step by step. It would be hard for pregnant adolescents to read the decision aid by themselves as they would either not read the information, read just a part of it, or not read at all as some cannot read or write. Tanzanians are reported to have a poor tendency of reading individually; that is, they have a poor reading culture [43].

In Tanzania, several decision aids have been used in clinics to provide family planning counseling to women. These include the World Health Organization Decision Making Tools [34, 44], Balanced Counseling Strategy [45], flipcharts, and counseling cards. However, these tools are only designed to support healthcare providers in family planning counseling and are not meant to help make informed decisions. Other family planning interventions implemented in Tanzania include the Chaguo la Maisha project by the Management and Development for Health and Pathfinder International [46]. This project provided essential information and accessibility of various family planning methods to women of reproductive age using community mobilizers. However, the intervention had an insufficient strategy of meeting adolescents below 18 years of age and pregnant adolescents to help them make an informed decision and prevent subsequent unintended pregnancies. Tanzania also had an effective mobile job-aid used to provide family planning counseling. However, the aid was only designed to support community health workers in providing family planning services but not to help mothers in making a family planning decision [46]. To our knowledge, the postpartum Green Star family planning decision aid that we developed in this study is the first pregnant adolescents-centered material for those who will attend antenatal care services.

Currently, health providers in Tanzania conduct group family planning counseling using the World Health Organization Family Planning Handbook [42] with the occasional use of flipcharts and a family planning medical eligibility criteria wheel as additional aids [44]. The family planning counseling offered to women is mainly provider-dependent. In contrast, the *postpartum Green Star family planning decision aid* will help provide essential information about the available long-acting reversible contraceptive methods. The tool will also ensure the information is objectively delivered to each pregnant adolescent.

The findings from this feasibility study suggest that the postpartum Green Star family planning decision aid is practical, useful, and acceptable for educating pregnant adolescents in Tanzania about long-acting reversible contraception methods that are available immediately after childbirth. The study participants proposed some modifications to improve the quality of the decision aid, including rearranging the flow of the contents, removing one of the sections, and changing some of the Kiswahili words to remove ambiguity. The decision aid should also be delivered and explained by healthcare providers to pregnant adolescents rather than letting them read the decision aid by themselves. This will ensure clear comprehension of information to simplify the decision-making process. Adding content that corrects family planning myths and misconceptions or omitting unimportant information is expected to enhance further the practicality, usefulness, and acceptability of the decision aid.

### Strengths and limitations of the study

For strengths, the study included pregnant adolescents and healthcare providers to assess the practicality, usefulness, and acceptability of the *postpartum Green Star family planning decision aid*. The study participants were also purposively selected to ensure the integrity and appropriateness of the decision aid.

The study participants were from a single hospital (i.e., Amana District Hospital), one of the several district hospitals in Dar es Salaam. Therefore, the present findings may not be sufficient to be generalized to other settings or groups of adolescents in Tanzania.

## Conclusion

Both pregnant adolescents and healthcare providers perceived the *postpartum Green Star family planning decision aid* as feasible in its practicality, usefulness, and acceptability. Family planning counseling tools with evidence-based information can support pregnant adolescents in improving their knowledge about family planning and improving informed, voluntary decisions. Further research is needed to assess the effects of the postpartum Green Star family planning decision aid on the choice of the long-acting reversible contraception methods among pregnant adolescents in Tanzania.

## Data Availability

Not applicable.
